# Effects of Diethylstilbestrol on the Structure and Function of the Spleen in Male Golden Hamsters

**DOI:** 10.3390/toxics13050397

**Published:** 2025-05-15

**Authors:** Jian Li, Ruiping Xu, Qingwei Wang, Xue Bai, Yanhua Su, Yaoxing Chen, Jing Cao

**Affiliations:** 1 Key Laboratory of Embryo Development and Reproductive Regulation Anhui Province, College of Biology and Food Engineering, Fuyang Normal University, Fuyang 236037, China; lijian800702@126.com; 2 Laboratory of Anatomy of Domestic Animals, National Key Laboratory of Veterinary Public Health and Safety, College of Veterinary Medicine, China Agricultural University, Haidian, Beijing 100193, China; xrp15237375652@163.com (R.X.);; 3 College of Veterinary Medicine, Yunnan Agricultural University, Panlong, Kunming 650201, China

**Keywords:** diethylstilbestrol, estrogen receptor, oxidative stress, spleen, male golden hamster

## Abstract

With industrial development, endocrine-disrupting chemicals have continued to accumulate in the environment, attracting growing attention due to their potential effects on biological health. The reproductive toxicity of diethylstilbestrol (DES), a synthetic estrogen widely present in the environment, is widely documented; however, studies on its effects on the immune system remain limited. In this study, adult male golden hamsters were subcutaneously administered varying doses of DES (0, 0.01, 0.1, and 1.0 mg/kg) for seven consecutive days to assess its immunomodulatory impact on peripheral blood and the spleen. We found that the DES treatment significantly reduced spleen index, white pulp area, and splenic lymphocyte proliferation while increasing caspase-3-positive apoptotic cells and inducible nitric oxide synthase expression. In peripheral blood, DES induced a dose-dependent suppression of lymphocyte proliferation, with lipopolysaccharide- and concanavalin A-stimulated proliferation reduced by 47.68–71.76% and 44.23–72.7%, respectively. Concurrently, DES significantly downregulated the pro-inflammatory cytokines IL-2 and IFN-γ (*p* < 0.01) while upregulating the anti-inflammatory cytokines IL-4 and IL-10 (*p* < 0.01). Furthermore, DES treatment impaired antioxidant defenses, decreasing the activity of superoxide dismutase, glutathione peroxidase, and catalase while elevating malondialdehyde levels. Notably, DES led to the upregulation of G protein-coupled estrogen receptor and estrogen receptor α at both transcriptional and protein levels, whereas estrogen receptor β mRNA expression increased despite a decline in protein levels. This study provides critical experimental evidence elucidating the immunoregulatory effects of endocrine-disrupting environmental estrogens.

## 1. Introduction

The potential health risks posed by environmental pollutants have garnered increasing global attention. International environmental organizations have classified certain chemicals as endocrine-disrupting chemicals (EDCs), a group of compounds capable of interfering with hormonal regulation and eliciting a wide range of biological effects, including both reversible and irreversible physiological changes [1]. EDCs primarily originate from industrial and domestic activities, with common pollutants including additives in organic chemicals and plastic products [2]. Based on their mechanisms of action, EDCs can be further categorized into environmental estrogens, androgens, and thyroid disruptors [3]. Of these, a substantial number exhibit estrogenic activity, including nonylphenol, phthalates, bisphenol A (BPA), and diethylstilbestrol (DES) [4]. DES, a synthetic estrogen first synthesized by Charles Dodds in 1938, has been of particular concern due to its potent biological activity and steroid hormone-like properties, positioning it as a key member of the EDC family [5]. Structurally analogous to endogenous estrogens, DES exhibits high affinity for estrogen receptors, enabling it to mimic nearly all natural estrogen functions. Consequently, from the mid-1940s to the early 1970s, DES was widely utilized in the medical field to treat female infertility, dysfunctional uterine bleeding, and gonadal dysgenesis [6]. It was also extensively applied in animal husbandry to enhance protein synthesis, increase milk production, and reduce fat deposition, thereby facilitating rapid growth in livestock [7,8].

Despite its extensive use in clinical medicine and animal husbandry, the long-term administration of DES has been associated with severe adverse effects, including reproductive toxicity and carcinogenicity [9]. Although the clinical application of DES has been banned, it remains a prototypical model compound for studying the immunotoxicological effects of environmental estrogen exposure [10]. Emerging evidence suggests that estrogens modulate immune cell differentiation and cytokine secretion, thereby exerting direct and indirect effects on peripheral immune organs [11,12]. As the largest reservoir of lymphocytes, the spleen plays crucial roles in antigen presentation, antibody production, and oxidative stress regulation [13]. Previous studies have reported that DES disrupts immune cell function by binding to its receptors, impairing cellular proliferation, cytokine secretion, and antigen presentation capacity [14]. These findings indicate that estrogen signaling pathways may regulate immune homeostasis by modulating the balance between cell proliferation and apoptosis [15]. While most research has focused on DES-induced disruptions in the female reproductive system, its immunotoxic effects on male-specific immune organs remain poorly understood. To bridge this gap, this study utilizes the male golden hamster as an experimental model, utilizing histological, molecular, and immunohistochemical approaches to elucidate the impact of DES on splenic structure and function. Our findings provide critical experimental insights into the immunotoxicological assessment of environmental estrogens.

## 2. Materials and Methods

### 2.1. Animal Management and Sample Collection

Seven-week-old male golden hamsters with a mean body weight of 117.5 ± 7.5 g were procured from Beijing Vital River Laboratory Animal Technology Co., Ltd. (Beijing, China). These animals were then randomly divided into four experimental groups, each consisting of ten male golden hamsters (*n* = 10). Following a seven-day acclimation period, the hamsters were divided into four experimental groups: the control group (0 mg/kg, olive oil), the low-dose group (0.01 mg/kg DES), the medium-dose group (0.1 mg/kg DES), and the high-dose group (1.0 mg/kg DES). DES (Sigma, Darmstadt, Germany) was dissolved in olive oil (MedChemExpress, Shanghai, China) and administered through subcutaneous injection in the neck region once daily for seven consecutive days. Animals were provided standard chow three times daily and had unrestricted access to water under controlled environmental conditions (temperature: 23–30 °C, humidity: 65–70%) and natural light cycles. Husbandry and disease prevention procedures adhered strictly to standard laboratory animal care guidelines. After the experiment, body weight measurements were taken prior to euthanasia. Under anesthesia, blood samples were collected via cardiac puncture, allowed to coagulate for 20 min at ambient temperature, and subsequently spun at 3000 rpm for 20 min at a chilled 4 °C to obtain the supernatant. Spleens were surgically removed, weighed, and prepared for subsequent analyses by fixing in 4% paraformaldehyde–phosphate-buffered saline (pH 7.4, Beyotime, Shanghai, China) for histological evaluation or stored at −80 °C for molecular studies. All experimental procedures were approved by the laboratory animal welfare and ethics committee of Fuyang Normal University, under the approval number FYNU2022AP011.

### 2.2. Hematoxylin and Eosin Staining

Spleen tissues preserved in 4% paraformaldehyde–phosphate-buffered saline (pH 7.4) underwent sequential ethanol dehydration, followed by xylene clearing and paraffin embedding. Thin serial sections (5 μm) were cut, deparaffinized, rehydrated, and stained with Ehrlich’s hematoxylin (Beyotime, Shanghai, China) for 20 min. Differentiation was performed using acid alcohol, followed by bluing in running tap water and counterstaining with 1% eosin. After being progressively dehydrated in a series of ethanol solutions and cleared with xylene, the sections were mounted with neutral balsam (Beyotime, Shanghai, China). The relative areas of the periarteriolar lymphoid sheath (PALS), splenic nodules (SNs), white pulp (WP), and red pulp (RP) were quantified using a microscope (Olympus BX51, Tokyo, Japan) with an image acquisition system and analyzed with Image-Pro Plus 6.0 software. For each tissue section, the areas of the PALS, SN, and RP, and the cross-section of the spleen were measured. The relative area of each region was calculated by dividing the area of that region by the cross-sectional area of the spleen and expressed as a percentage. Specifically, the relative area of the PALS was calculated using the following formula: relative area of PALS = (area of PALS/cross-sectional area of the spleen) × 100%. The relative area of SN was determined in the same way. The white pulp (WP) area was defined as the sum of PALS and SN areas, and its relative area was calculated accordingly. The relative area of RP was calculated as follows: relative area of RP = (area of RP/cross-sectional area of the spleen) × 100%.

### 2.3. Immunohistochemical Staining

Paraffin-embedded spleen sections underwent deparaffinization, rehydration, and antigen retrieval in 0.01 M citrate buffer (pH 6.0). Endogenous peroxidase activity was quenched with 3% hydrogen peroxide, followed by blocking with 5% goat serum. The sections were then incubated overnight at 4 °C with primary antibodies, including mouse anti-human Proliferating Cell Nuclear Antige (PCNA) monoclonal antibody (1:2000, ZSGB-BIO, Beijng, China), rabbit anti-mouse caspase-3 polyclonal antibody (1:200, ZSGB-BIO, Beijng, China), rabbit anti-mouse inducible nitric oxide synthase (iNOS) polyclonal antibody (1:200, ZSGB-BIO, Beijng, China), mouse anti-estrogen receptor α (ERα) monoclonal antibody (1:100, Enzo Life Sciences, Inc., Farmingdale, NY, USA), rabbit anti-mouse estrogen receptor beta (ERβ) polyclonal antibody (1:300, Invitrogen, Waltham, MA, USA), and rabbit anti-mouse G protein-coupled estrogen receptor (GPER) polyclonal antibody (1:200, Novus Biologicals, Centennial, CO, USA). On the next day, the sections were warmed to room temperature, rinsed with PBS, and then incubated for two hours with biotinylated secondary antibodies: goat anti-rabbit IgG (for caspase-3, iNOS, GPER, and ERβ; ZSGB-BIO, Beijng, China), goat anti-mouse IgG (for ERα; Kangwei Century, Beijng, China), or horse anti-mouse IgG (for PCNA; ZSGB-BIO, Beijng, China). After washing, streptavidin–horseradish peroxidase (1:1000, Vector Laboratories, Newark, CA, USA) was applied, and incubation was performed at room temperature for 1.5 h, followed by rinsing the sections with TBST. Visualization was achieved using 3,3′-diaminobenzidine (DAB, Beyotime, Shanghai, China) substrate with hematoxylin counterstaining. The sections were dehydrated, subjected to xylene for clearing, and then mounted using neutral balsam (Beyotime, Shanghai, China). Immunoreactive cells were visualized and quantified using an Olympus BX51 microscope (Olympus, Tokyo, Japan).

### 2.4. Assessment of Lymphocyte Proliferation

In accordance with the previous literature, the MTT assay was utilized to evaluate the proliferative response of peripheral blood and splenic lymphocytes to concanavalin A (Con A) and lipopolysaccharide (LPS) [16]. A suspension of splenocytes as single cells was obtained by mechanically dissociating the tissue using a nylon mesh. Peripheral blood and splenic single-cell suspensions were mixed with lymphocyte separation medium at a 1:1 ratio and subjected to density gradient centrifugation to isolate lymphocytes, followed by three washes with RPMI-1640 (Gibco, Thermo Fisher Scientific, Waltham, MA, USA) complete medium. The viability of cells was assessed using the trypan blue exclusion method, and the cell concentration was set to 1 × 10^6^ cells/mL. Subsequently, 190 μL of the prepared suspension was dispensed into each well of a 96-well plate, followed by the addition of 10 μL of either Con A (at concentrations of 5, 10, or 20 μg/mL) or LPS (at 5, 10, or 20 μg/mL). Three technical replicates and blank controls were included for each condition. After incubation in a CO₂ incubator (Thermo Fisher Scientific, Waltham, MA, USA) for 44 h, MTT was introduced and incubated for 4 h. After this, a 10% SDS solution was added, and the mixture was incubated at 37 °C for an additional 2 h. The plates were left to equilibrate at room temperature for 20 min, after which the optical density (OD) at 570 nm was measured to assess lymphocyte proliferation activity.

### 2.5. Quantification of Peripheral Blood Cytokines

The concentrations of cytokines in peripheral blood, including IL-2, IL-4, IL-10, and IFN-γ, were quantified with enzyme-linked immunosorbent assay (ELISA) kits supplied by Jiangsu Meimian Industrial Co. (Nanjing, China), following the instructions outlined by the manufacturer. Briefly, whole blood was collected from the orbital sinus and centrifuged at 3000 rpm for 20 min to obtain the serum. The serum samples were added to 96-well plates pre-coated with specific monoclonal antibodies, followed by incubation with biotinylated detection antibodies and streptavidin–HRP. Standard curves were constructed for each cytokine, and sample concentrations were calculated accordingly.

### 2.6. Measurement of Antioxidant Enzyme Activity

The enzymatic activities of superoxide dismutase (SOD) and glutathione peroxidase (GSH-Px), total antioxidant capacity (T-AOC), and the level of malondialdehyde (MDA) were determined in spleen and peripheral blood serum homogenates using commercial colorimetric assay kits (Nanjing Jiancheng Bioengineering Institute, Nanjing, China), following the manufacturer’s instructions. For blood samples, whole blood was collected from the orbital sinus and centrifuged at 3000 rpm for 10 min to separate the serum. For spleen tissue analysis, the samples were weighed, homogenized in ice-cold normal saline (1:9, *w*/*v*), and centrifuged at 3000 rpm for 10 min at 4 °C to obtain supernatants. All subsequent procedures were carried out strictly in accordance with the guidelines provided by the manufacturer.

### 2.7. Quantitative Real-Time PCR (RT-qPCR)

RT-qPCR was conducted using a LightCycler^®^ 480 system (Roche Applied Science, Penzberg, Germany) with a 20 μL reaction mixture. This included 10 μL SYBR Green PCR Master Mix (Q121-02, Vazyme, Nanjing, China), 2 μL cDNA, 0.4 μL forward primer, 0.4 μL reverse primer, and 7.2 μL nuclease-free water. Primers were specifically designed with Primer Premier 5.0 and synthesized by Sangon Biotech (Shanghai, China) (Table 1). The relative expression levels of target gene mRNA were determined utilizing the 2^−ΔΔCt^ method.

### 2.8. Western Blot Analysis

The splenic tissues were first homogenized and centrifuged at 12,000 rpm for 10 min; the supernatant was then collected for protein quantification and adjusted to a final concentration of 2.5 μg/μL. A total of 100 μL of protein lysate was combined with 5× sodium dodecyl sulfate–polyacrylamide gel electrophoresis loading buffer and heated at 99 °C for 10 min. Proteins were isolated using 10% resolving gels and 12% stacking gels, with electrophoresis performed at 60 V for 30 min, which then increased to 120 V for 60 min. Subsequently, target protein bands were transferred onto a PVDF membrane under a steady current of 200 mA for 1.5 h. After TBST washing, the membrane was blocked in 5% BSA blocking solution for 60 min. Primary antibodies, including mouse monoclonal anti-ERα (1:1500, Enzo Life Sciences, Inc., Farmingdale, NY, USA), rabbit polyclonal anti-ERβ (1:2000, Invitrogen, Waltham, MA, USA), rabbit polyclonal anti-GPER (1:1000, Novus Biologicals, Centennial, CO, USA), and mouse monoclonal anti-β-actin (1:10,000, Proteintech, Rosemont, IL, USA), were incubated overnight at 4 °C. HRP-conjugated goat anti-rabbit IgG (for GPER and ERβ, Kangwei Century, Beijng, China) and HRP-conjugated goat anti-mouse IgG (for ERα, Kangwei Century, Beijng, China) were subsequently added and incubated at room temperature for 1.5 h, followed by three washes with TBST. Protein signals were detected using enhanced chemiluminescence (ECL) and visualized with a gel imaging system (Scientific Imaging Manufacturing Corporation, San Jose, CA, USA). Protein band intensities were quantified using ImageJ 1.53 software (National Institutes of Health, Bethesda, MD, USA), and graphical representations were generated with GraphPad Prism 8.4.3 (GraphPad Software Inc., San Diego, CA, USA).

### 2.9. Statistical Analysis

Statistical analysis was carried out using IBM SPSS Statistics 20 (SPSS Inc., Chicago, IL, USA). To compare the different groups, a one-way analysis of variance (ANOVA) was employed, followed by post hoc least significant difference (LSD) tests for multiple pairwise comparisons. The data are expressed as the mean ± standard error of the mean (SEM), and statistical significance was considered at *p* < 0.05.

## 3. Results

### 3.1. DES Reduced Body Weight and Spleen Index in Male Golden Hamsters

After a 7-day treatment with 0.01 and 0.1 mg/kg DES, no significant changes in body weight were detected in male golden hamsters when compared to the control group (*p* > 0.05), whereas a significant reduction was noted in the 1.0 mg/kg group (*p* < 0.01). Additionally, the average daily gain (ADG) was reduced by 30.21% (*p* > 0.05), 46.99% (*p* < 0.05), and 72.38% (*p* < 0.01) in the 0.01, 0.1, and 1.0 mg/kg groups, respectively, compared with the controls. The spleen index in the 1.0 mg/kg treatment group was notably reduced by 23.48% compared to the control group (*p* < 0.05, Table 2).

### 3.2. DES Altered Splenic Histological Architecture in Male Golden Hamsters

**Figure 1:**
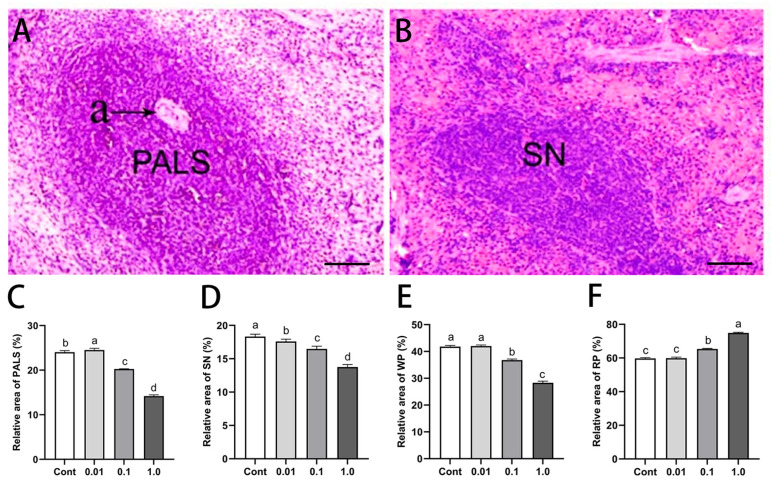
Effects of DES on splenic histomorphology in male golden hamsters: (**A**,**B**) Representative hematoxylin and eosin staining of the periarteriolar lymphoid sheath (PALS) and splenic nodule (SN) regions in the spleen of adult male golden hamsters. The artery is denoted by “a”; scale bar = 100 μm. (**C**–**F**) Quantitative analysis of the relative area of the PALS, SN, white pulp (WP), and red pulp (RP). Different letters (e.g., a, b, c, d) indicate statistically significant differences between groups (*p* < 0.05). Groups sharing at least one common letter are no different from each other (*p* > 0.05). Data are presented as mean ± SEM.

HE staining revealed dose-dependent histopathological alterations in the spleen following DES exposure (Figure 1). As the DES concentration increased, the relative areas of the PALS, SN, and WP progressively decreased, whereas the RP expanded. Compared with the control group, PALS in the 0.1 and 1.0 mg/kg groups exhibited nuclear condensation, fragmentation, necrosis, and degeneration, with the relative areas reduced by 14.46% (*p* < 0.05) and 41.24% (*p* < 0.01), respectively. Similarly, in the 1.0 mg/kg group, the SN and WP areas were notably reduced by 25.21% (*p* < 0.05) and 34.30% (*p* < 0.01), respectively. Conversely, the RP area increased by 8.96% (*p* < 0.05) and 23.91% (*p* < 0.01) in the 0.1 and 1.0 mg/kg groups, respectively, compared with the control group.

### 3.3. DES Inhibited Splenocyte Proliferation While Inducing Apoptosis and iNOS Expression in Male Golden Hamsters

**Figure 2:**
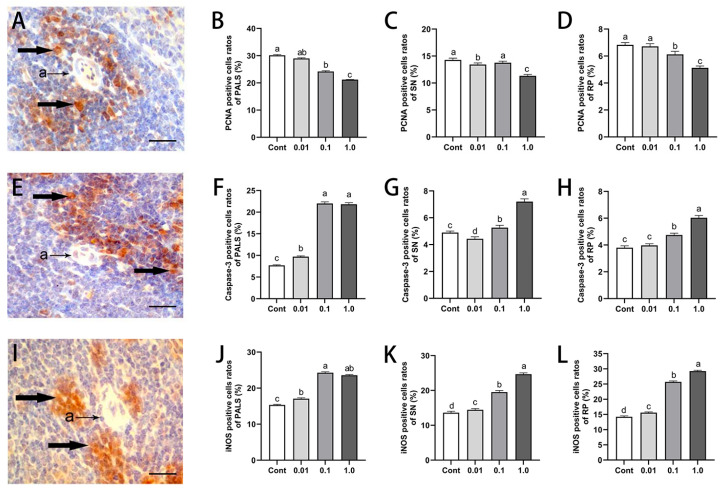
Effects of DES on splenic cell proliferation and apoptosis in male golden hamsters: (**A**–**D**) Immunohistochemical staining and quantification of proliferating cell nuclear antigen (PCNA)-positive cells in the spleen of adult male golden hamsters. (**E**–**H**) Immunohistochemical staining and quantification of caspase-3-positive cells in the spleen of adult male golden hamsters. (**I**–**L**) Immunohistochemical staining and quantification of inducible nitric oxide synthase (iNOS)-positive cells in the spleen of adult male golden hamsters. Arrows indicate positively stained cells, while “a” represents the artery. Scale bar = 30 μm. Different letters (e.g., a, b, c, d) indicate statistically significant differences between groups (*p* < 0.05). Groups sharing at least one common letter are no different from each other (*p* > 0.05). Data are expressed as mean ± SEM.

The immunohistochemical analysis of PCNA, caspase-3, and iNOS revealed distinct immunopositive cell distributions across the PALS, SN, and RP (Figure 2). PCNA and caspase-3 signals were predominantly localized in the nucleus, whereas iNOS expression was mainly confined to the cytoplasm. DES exposure led to a dose-dependent reduction in PCNA-positive cell rates, whereas caspase-3 and iNOS expression exhibited a progressive increase with escalating DES concentrations. In comparison to the control group, the 1.0 mg/kg DES-treated animals exhibited a significant decline in PCNA-positive cell rates within the PALS (30.28%, *p* < 0.01), SN (21.36%, *p* < 0.05), and RP (25.36%, *p* < 0.05). In contrast, caspase-3- and iNOS-positive cell rates were markedly elevated, with respective increases of 182.1% and 52.75% (*p* < 0.01) in the PALS, 42.71% and 103.07% (*p* < 0.05) in the SN, and 56.86% and 81.32% (*p* < 0.01) in the RP. Notably, low-dose DES treatment (0.01 mg/kg and 0.1 mg/kg) did not significantly affect PCNA expression across the analyzed regions (*p* > 0.05), whereas a notable increase of 25.4% (*p* < 0.05) in caspase-3-positive cell rates was noted in the PALS of the 0.01 mg/kg group.

### 3.4. DES Inhibited Proliferation of Splenic and Peripheral Blood Lymphocytes in Male Golden Hamsters

**Figure 3:**
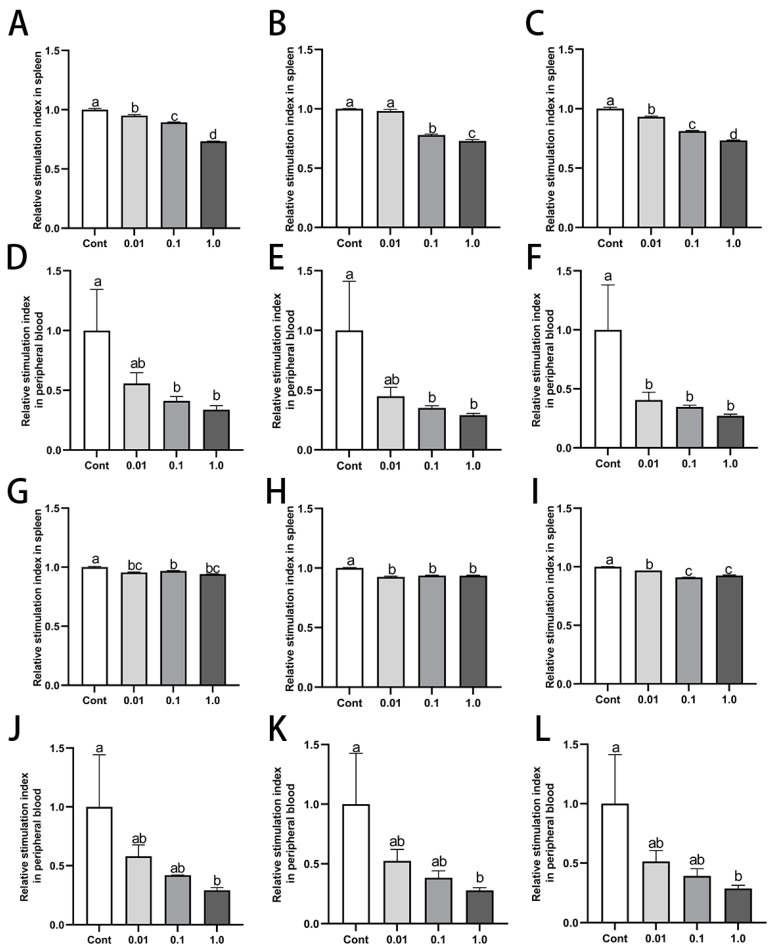
Effects of DES on the proliferative activity of splenic and peripheral blood lymphocytes in male golden hamsters: (**A**–**C**) Proliferative response of splenic lymphocytes to concanavalin A (Con A, 5, 10, and 20 mg/kg) following DES treatment. (**D**–**F**) Proliferative response of peripheral blood lymphocytes to Con A (5, 10, and 20 mg/kg) following DES treatment. (**G**–**I**) Proliferative response of splenic lymphocytes to lipopolysaccharide (LPS, 5, 10, and 20 mg/kg) following DES treatment. (**J**–**L**) Proliferative response of peripheral blood lymphocytes to LPS (5, 10, and 20 mg/kg) following DES treatment. Different letters (e.g., a, b, c, d) indicate statistically significant differences between groups (*p* < 0.05). Groups sharing at least one common letter are no different from each other (*p* > 0.05). Data are expressed as mean ± SEM.

DES treatment notably inhibited the proliferative activity of both splenic and peripheral blood lymphocytes in male golden hamsters (Figure 3). In comparison to the control group, the splenic and peripheral blood lymphocytes of the groups treated with 0.1 mg/kg and 1.0 mg/kg DES exhibited a marked reduction in lymphocyte proliferation upon Con A and LPS stimulation (*p* < 0.05). The 1.0 mg/kg group showed the most pronounced inhibitory effect. Specifically, in the 1.0 mg/kg DES group, the stimulation index of spleen lymphocytes decreased by 27.97% (*p* < 0.01) and 6.19% (*p* > 0.05); 28.63% (*p* < 0.01) and 6.43% (*p* < 0.05); and 27.08% (*p* < 0.01) and 7.76% (*p* < 0.05) after stimulation with 5 μg/mL, 10 μg/mL, and 20 μg/mL of Con A and LPS, respectively. Similarly, for peripheral blood lymphocytes in the 1.0 mg/kg DES group, the stimulation index decreased by 66.22% (*p* < 0.05) and 71.76% (*p* < 0.05); 70.94% (*p* < 0.05) and 72.09% (*p* < 0.01); and 72.70% (*p* < 0.05) and 71.15% (*p* < 0.05) after stimulation with 5 μg/mL, 10 μg/mL, and 20 μg/mL of Con A and LPS, respectively.

### 3.5. DES Modulated Peripheral Cytokine Profiles in Male Golden Hamsters

**Figure 4:**
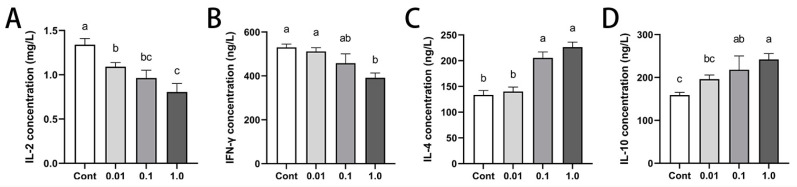
Effects of DES on peripheral blood cytokine levels in male golden hamsters: (**A**–**D**) Serum concentrations of IL-2, IFN-γ, IL-4, and IL-10. Different letters (e.g., a, b, c) indicate statistically significant differences between groups (*p* < 0.05). Groups sharing at least one common letter are no different from each other (*p* > 0.05). Data are expressed as mean ± SEM.

To better understand the effects of immunomodulation of DES on immune homeostasis and the splenic microenvironment, we quantified the expression levels of Th1/Th2 cytokines (Figure 4). Increasing DES doses led to a dose-dependent decline in Th1 cytokines, with IL-2 and IFN-γ levels significantly reduced, particularly with the 1.0 mg/kg treatment (*p* < 0.01). IL-2 levels decreased by 18.57% (*p* < 0.05), 28.08% (*p* < 0.01), and 39.95% (*p* < 0.01) in the 0.01, 0.1, and 1.0 mg/kg groups, respectively. Similarly, IFN-γ levels exhibited a significant reduction of 26.17% (*p* < 0.01) exclusively in the 1.0 mg/kg group. In contrast, the Th2 cytokines IL-4 and IL-10 exhibited a dose-dependent increase, reaching statistical significance at 1.0 mg/kg (*p* < 0.01). Specifically, IL-4 levels increased by 54.01% and 69.57% (*p* < 0.01) in the 0.1 and 1.0 mg/kg groups, respectively, while IL-10 levels rose by 37.07% (*p* < 0.05) and 53.14% (*p* < 0.01) at the same doses.

### 3.6. DES Impaired Antioxidant Capacity in Spleen and Peripheral Blood of Male Golden Hamsters

**Figure 5:**
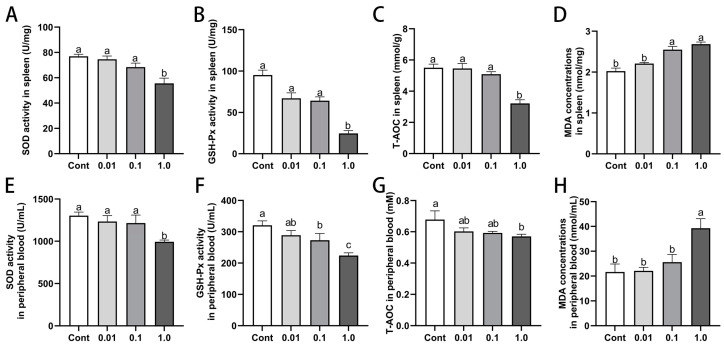
Effects of DES on antioxidant capacity in spleen and peripheral blood of male golden hamsters: (**A**–**D**) Levels of superoxide dismutase (SOD), glutathione peroxidase (GSH-Px), total antioxidant capacity (T-AOC), and malondialdehyde (MDA) in the spleen. (**E**–**H**) Levels of SOD, GSH-Px, T-AOC, and MDA in peripheral blood. Different letters (e.g., a, b, c) indicate statistically significant differences between groups (*p* < 0.05). Groups sharing at least one common letter are no different from each other (*p* > 0.05). Data are expressed as mean ± SEM.

DES exposure led to a dose-dependent decline in the antioxidant capacity of both the spleen and peripheral blood (Figure 5). The activities of SOD, GSH-Px, and T-AOC progressively decreased with increasing DES doses, with the most pronounced reduction observed at 1.0 mg/kg (*p* < 0.05). Specifically, in comparison to the control group, SOD activity was reduced by 34.22% (*p* < 0.05) in the spleen and 23.75% (*p* < 0.01) in peripheral blood, while GSH-Px activity decreased by 71.92% (*p* < 0.01) and 30.14% (*p* < 0.01), respectively. Similarly, T-AOC levels declined by 39.58% (*p* < 0.05) in the spleen and 15.91% (*p* < 0.05) in peripheral blood. In contrast, MDA, which serves as an indicator of lipid peroxidation, exhibited significant increases of 32.56% (*p* < 0.05) in the spleen and 81.26% (*p* < 0.01) in peripheral blood.

### 3.7. DES Modulated Estrogen Receptor Expression in the Spleen of Male Golden Hamsters

**Figure 6:**
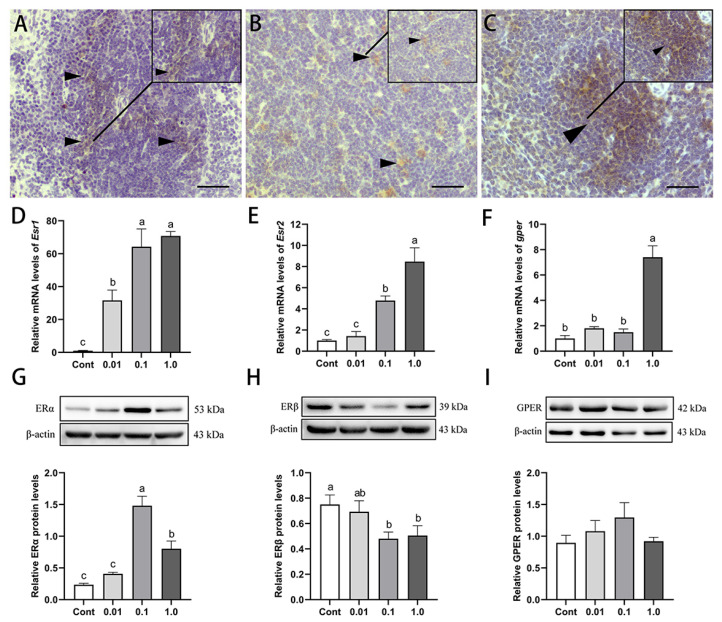
Effects of DES on estrogen receptor expression in the spleen of adult male golden hamsters: (**A**–**C**) Immunohistochemical localization of ERα, ERβ, and GPER in the splenic tissue. Triangles indicate estrogen receptor-positive cells. Scale bar = 50 μm. (**D**–**F**) mRNA expression levels of Esr1, Esr2, and Gper in the spleen. (**G**–**I**) Protein expression levels of ERα, ERβ, and GPER in the spleen. Different letters (e.g., a, b, c) indicate statistically significant differences between groups (*p* < 0.05). Groups sharing at least one common letter are no different from each other (*p* > 0.05). Data are expressed as mean ± SEM.

Exposure to DES altered the expression of estrogen receptors in the spleens of adult male golden hamsters (Figure 6). Immunohistochemical analysis revealed the positive localization of ERα, ERβ, and GPER within the periarteriolar lymphoid sheath and splenic follicles (Figure 6A–C). Esr1, Esr2, and gper mRNA levels exhibited a dose-dependent increase following DES treatment; they were significantly elevated by 69.75% (*p* < 0.01), 7.46% (*p* < 0.01), and 6.40% (*p* < 0.01), respectively, in the 1.0 mg/kg DES group when compared to the control group. Western blot analysis further revealed that ERα protein levels peaked in the 0.1 mg/kg group, showing a striking 527.60% increase (*p* < 0.01) relative to the control group, whereas ERβ expression was most reduced in this group, with a 35.96% decline (*p* < 0.05). No notable changes in GPER protein expression were detected among the treatment groups (*p* > 0.05).

## 4. Discussion

With the advancement of industrialization, increasing attention has been directed toward the impact of environmental estrogens on animal health. As a crucial peripheral immune organ, the spleen plays a pivotal role in immune function, with its developmental status serving as a key indicator of systemic immunity. The PALS within the splenic white pulp is predominantly composed of T cells and expands during cellular immune responses [17,18]. In contrast, the SN, a B cell-enriched region, remains relatively limited in size under normal physiological conditions but undergoes significant expansion during humoral immune activation [17]. Our study demonstrated that DES exposure resulted in reduced body weight gain, a decreased spleen index, and a contraction of both the PALS and SN areas. Additionally, the percentage of PCNA-positive cells decreased in a manner that was dependent on the dose, with the most pronounced reduction observed in the PALS region, while the proportion of caspase-3-positive apoptotic cells increased correspondingly. These findings suggest that DES exerts a dual immunosuppressive effect on both cellular and humoral immunity, potentially by disrupting the balance between T lymphocyte proliferation and apoptosis in the spleen.

iNOS catalyzes the conversion of L-arginine into NO, exhibiting a bidirectional regulatory role in inflammation and oxidative stress [19]. Research has shown that inflammatory stimuli, such as cytokines and endotoxins, can significantly upregulate iNOS expression via the activation of the NF-κB and MAPK signaling pathways [20]. Under physiological conditions, NO exerts protective effects by mediating immune defense and vasodilation; however, excessive production of NO can interact with reactive oxygen species (ROS), leading to the formation of peroxynitrite (ONOO⁻), which triggers oxidative stress cascades that exacerbate tissue damage and promote the progression of inflammatory diseases [21]. Notably, oxidative stress can further enhance iNOS expression through positive feedback mechanisms involving NF-κB and other signaling pathways, thereby establishing a self-perpetuating cycle of inflammation and oxidative stress [22,23]. Moreover, DES-induced iNOS upregulation may disrupt immune homeostasis through a dual mechanism: at low concentrations, NO inhibits apoptosis, whereas at high concentrations, it promotes cell death by disrupting mitochondrial membrane potential (MMP) [24,25,26,27,28,29]. This mechanistic dichotomy may explain the heightened apoptosis observed in the PALS region of the spleen, while the SN and RP regions exhibited greater resistance to DES-induced cytotoxicity.

To further investigate whether DES affects lymphocyte function, we assessed lymphocyte proliferation in peripheral blood and the spleen following stimulation with Con A and LPS. The results demonstrated a significant suppression of both T and B lymphocyte proliferation, with Con A-induced suppression of peripheral blood and splenic T lymphocytes being the most pronounced. These findings indicate that DES exerts a potent inhibitory effect on T lymphocyte-mediated cellular immunity in both peripheral blood and spleen. This observation aligns with previous reports suggesting that DES exposure disrupts the positive and negative selection of thymic T cells, thereby impairing T cell differentiation and function [30]. Additionally, DES has been shown to inhibit mitosis in murine lymphocytes at concentrations ranging from 10^−6^ to 10^−5^ M [15], while higher doses (30 mM) induce DNA strand breaks in lymphocytes, which may contribute to immune dysfunction and are implicated in the carcinogenic properties of DES [31]. Our study further revealed that DES treatment led to a reduction in Th1 cytokines (IL-2 and IFN-γ) while simultaneously causing an elevation in Th2 cytokines (IL-4 and IL-10), suggesting a shift toward Th2 differentiation and an imbalance in the Th1/Th2 ratio. Such dysregulation is closely associated with various pathological conditions, including Th1-dominant autoimmune diseases [32] and Th2-skewed allergic disorders [33]. These findings support the notion that DES modulates T cell differentiation through cytokine network regulation, though its precise mechanisms remain dose- and exposure-dependent and warrant further investigation.

Under normal physiological conditions, the body maintains redox homeostasis through an intricate antioxidant enzyme system, featuring SOD, CAT, and GSH-Px, which collectively neutralize ROS. However, endogenous metabolic imbalances or exposure to exogenous toxicants can result in excessive ROS accumulation, triggering oxidative stress [34] and resulting in lipid peroxidation, DNA damage, and protein oxidation [35]. While moderate ROS levels play a crucial role in immune regulation, excessive ROS can suppress T lymphocyte proliferation, disrupt T cell receptor signaling, and impair immune responses [36]. Oxidative stress is considered a crucial factor contributing to the immunotoxicity induced by DES. For instance, BPA exposure has been shown to elevate ROS levels in crayfish; diminish the activities of key antioxidant enzymes such as SOD, peroxidase, and CAT; and compromise antimicrobial immunity [37]. Similarly, zebrafish embryos exposed to BPA and nonylphenol exhibit increased ROS and nitric oxide synthase activity, along with the upregulated expression of IFN-γ, IL-1β, and IL-10 [38]. Consistent with these findings, our study demonstrated that high-dose DES exposure significantly reduced SOD, GSH-Px, and T-AOC activity in the peripheral blood and spleen of Mesocricetus auratus while markedly increasing MDA levels. These results suggest that DES exposure induces ROS accumulation and oxidative stress, potentially contributing to its immunosuppressive effects.

Studies have demonstrated that in thyroid cancer cells with high ERα expression, ERα can induce autophagy via the ROS-ERK signaling pathway to sustain cell survival. However, when ERα signaling becomes excessively activated or acts in concert with DNA damage signals, it triggers the p53-p21 axis, inhibiting the Cyclin–CDK complex, downregulating PCNA, and ultimately suppressing cell proliferation. This aligns with our findings, where elevated ERα expression is accompanied by a decrease in PCNA-positive cells [39,40,41,42]. Furthermore, ERβ is generally associated with antiproliferative and pro-apoptotic effects. Its downregulation weakens Bcl-2-mediated mitochondrial protection, increases mitochondrial membrane permeability, and activates the caspase-9/caspase-3 cascade, thereby enhancing apoptosis [43,44]. This is consistent with the observed upregulation of caspase-3-positive cells in our study. The slight increase in GPER expression suggests a potential compensatory mechanism for ERα/ERβ imbalance, although its precise role requires further validation using receptor knockout models. Notably, the Th1/Th2 cytokine imbalance observed in our study, characterized by decreased IL-2 and IFN-γ alongside elevated IL-4 and IL-10, correlates strongly with alterations in ERα/ERβ expression. This polarization may contribute to impaired antiviral and antitumor immunity while simultaneously increasing susceptibility to autoimmune diseases (e.g., rheumatoid arthritis and type 1 diabetes) and allergic disorders (e.g., asthma and allergic rhinitis) [45,46].

## 5. Conclusions

DES exerts immunosuppressive effects in male golden hamsters by binding to ERα in the spleen, leading to a decline in splenic antioxidant capacity and lymphocyte proliferative activity. This disruption induces a Th1/Th2 imbalance and enhances apoptosis, ultimately altering splenic tissue architecture and impairing immune homeostasis. These results offer valuable perspectives on the immunotoxic mechanisms of DES and highlight its possible effects on immune regulation due to endocrine-disrupting chemical exposure.

## Figures and Tables

**Table 1:** Sequences of primers used for PCR.

Genes	Primer Sequence (5′-3′)	Produce Size (bp)	Accession No.
*Esr1*	CAGCAGCAGCATCGTCGTCTG AGCATCCAACATCTCCAGCAACAG	147	NM_001281325.1
*Esr2*	TAGAACACACCTTGCCTGGGAG ACATCCTTCGCACGACCAGA	170	XM_013115381.2
*gper*	TTCCGCACCAAGCACCAT AGCCACTGCACCTCTCTGACA	149	XM_013120473.2
*β-actin*	GGCTCCCAGCACCATGAA GCCACCGATCCACACAGAGT	70	NM_001281595.1

**Table 2:** The effects of DES on the body weight and spleen weight (X ± SEM).

DES Dose (mg/kg·BW)	Initial Weight (g)	Final Weight (g)	Average Daily Gain (g)	Spleen Index (%)
control	129.3 ± 1.467 ^a^	142.8 ± 0.630 ^a^	1.930 ± 0.195 ^a^	0.1120 ± 0.0035 ^a^
0.01	129.9 ± 1.941 ^a^	139.3 ± 1.378 ^ab^	1.347 ± 0.083 ^ab^	0.1291 ± 0.0103 ^a^
0.1	128.3 ± 1.263 ^a^	136.2 ± 2.845 ^ab^	1.023 ± 0.164 ^b^	0.0956 ± 0.0052 ^a^
1.0	128.6 ± 0.440 ^a^	132.3 ± 1.135 ^b^	0.533 ± 0.129 ^c^	0.0857 ± 0.0064 ^b^

Note: Lowercase letters in the upper right corner indicate statistically significant differences within the groups (*p* < 0.05). Different letters (e.g., a, b, c) denote significant differences between groups at a predefined significance level (e.g., *p* < 0.05), while groups sharing at least one letter are not significantly different from each other. Data are presented as mean ± SEM.

## Data Availability

The original contributions presented in this study are included in the article. Further inquiries can be directed to the corresponding author.
